# Effects of Postbiotics Derived from Guava (*Psidium guajava* L.) Leaf Extract Bioconverted by *Limosilactobacillus fermentum* on Renal Inflammation in Type 2 Diabetic Mice

**DOI:** 10.3390/nu17193084

**Published:** 2025-09-28

**Authors:** Nayoung Park, Heaji Lee, Choong-Hwan Lee, Yunsook Lim

**Affiliations:** 1 Department of Food and Nutrition, Kyung Hee University, 26 Kyunghee-Daero, Dongdaemun-Gu, Seoul 02447, Republic of Korea; nayoung1901@khu.ac.kr (N.P.); ji3743@khu.ac.kr (H.L.); 2 Department of Food Science and Nutrition, Hallym University, Chuncheon 24252, Republic of Korea; 3 Department of Bioscience and Biotechnology, Konkuk University, Seoul 05029, Republic of Korea; chlee123@konkuk.ac.kr

**Keywords:** type 2 diabetes mellitus, diabetic nephropathy, *Limosilactobacillus fermentum*, postbiotics, guava leaf (*Psidium guajava* L.), bioconversion

## Abstract

Background/Objectives: Diabetic nephropathy (DN) is a major complication of diabetes and a leading cause of end-stage renal disease, a condition associated with high mortality risks. Recently, supplementation with probiotics and postbiotics has been attracting attention. Especially, metabolites of natural products bioconverted by beneficial bacteria have emerged as a novel therapeutic intervention for metabolic diseases, including diabetes, due to the enhanced bioavailability of their metabolites. This study investigated the alleviating effects of metabolites derived from guava leaf extract bioconverted by *Limosilactobacillus fermentum* (GBL) on renal inflammation in type 2 diabetic mice. Methods: For this purpose, diabetes was induced in male C57BL/6J mice by a high-fat diet and streptozotocin injection (80 mg/kg BW) twice. Subsequently, mice with fasting blood glucose levels higher than 300 mg/dL were administered metabolites of *L. fermentum* (LF) (50 mg/kg BW/day) or guava leaf extract bioconverted by *L. fermentum* (GBL) (50 mg/kg BW/day) by oral gavage for 15 weeks. Results: GBL demonstrated potential in alleviating hyperglycemia-induced DN in diabetic mice. It markedly improved hyperglycemia, glucose tolerance, and morphological alterations, which might stem from activation of key regulators of energy metabolism. GBL uniquely reduced advanced glycation end products (AGEs) and suppressed nucleotide-binding oligomerization domain-like receptor family pyrin domain-containing 3 (NLRP3) inflammasome and nuclear factor kappa-light-chain-enhancer of activated B cells (NF-κB)-driven inflammatory pathways, which significantly alleviated oxidative stress and apoptosis. Conclusions: This highlights the distinct therapeutic efficacy of GBL in addressing DN, primarily through its effects on renal inflammation. Taken together, GBL can be used as a promising nutraceutical to mitigate hyperglycemia and its associated renal inflammation, thereby alleviating the progression of DN.

## 1. Introduction

Type 2 diabetes mellitus (T2DM) is a chronic metabolic disease with a steadily rising global burden, posing a significant challenge to public health systems [1]. Chronic hyperglycemia, a hallmark of diabetes, contributes to complications in multiple organs, including kidneys [2]. The kidney is particularly vulnerable to hyperglycemia-induced damage due to its dense microvascular architecture, continuous filtration of large volumes of blood, and direct exposure of glomeruli and renal tubules. This vulnerability exacerbates pathogenic processes of diabetic nephropathy (DN), a major microvascular complication and the leading cause of end-stage renal disease (ESRD), which accelerates structural and functional deterioration [3]. Furthermore, renal injury progresses irreversibly from glomerular hypertrophy and mesangial expansion to nephron loss, ultimately culminating in ESRD if timely interventions are not implemented [4]. Therefore, prevention of early renal damage is critical to mitigate long-term complications, underscoring the urgent need for targeted renoprotective strategies.

Despite available therapies, current interventions primarily focus on symptom control and offer limited efficacy in halting disease progression [5]. This underscores the need for novel therapeutic strategies targeting the molecular mechanisms underlying DN. The pathogenesis of DN is driven by chronic hyperglycemia-induced molecular cascades involving oxidative stress, inflammation, apoptosis, and energy dysregulation [6]. Hyperglycemia accelerates the formation of advanced glycation end products (AGEs), which bind to the receptor for AGEs (RAGE), activating redox-sensitive pathways that elevate reactive oxygen species (ROS) and suppress antioxidant defenses such as the nuclear factor erythroid 2-related factor 2 (Nrf2) signaling pathway [7,8]. Oxidative imbalance amplifies inflammatory responses through the activation of the nucleotide-binding oligomerization domain-like receptor family pyrin domain-containing 3 (NLRP3) inflammasome and nuclear factor kappa-light-chain-enhancer of activated B cells (NF-κB), promoting the release of proinflammatory cytokines [9]. The sustained oxidative-inflammatory environment triggers mitochondrial dysfunction and activates pro-apoptotic signaling via p53 and the Bcl–2–associated X protein (Bax)/B-cell lymphoma 2 (Bcl-2) axis, leading to caspase-3-mediated cell death [10,11]. Furthermore, impaired energy-sensing mechanisms, particularly the adenosine monophosphate-activated protein kinase (AMPK)/sirtuin 1 (SIRT1)/peroxisome proliferator-activated receptor-gamma coactivator 1-alpha (PGC-1α) axis, exacerbate oxidative stress and hinder mitochondrial biogenesis, creating a vicious cycle that accelerates renal damage [12]. These interlinked processes collectively contribute to histological changes, glomerular injury, proteinuria, and progressive loss of renal function, underscoring the need for strategies that can target multiple molecular pathways [13].

Recent studies have reported the use of various probiotics, such as *Lactobacillus*, *Bifidobacterium*, and *Eubacterium*, for managing chronic diseases. Among them, the *Lactobacillus* family has shown renoprotective effects by exerting antioxidant, anti-inflammatory, and antidiabetic activities [14,15]. These effects are largely attributed to postbiotics—bioactive metabolites produced during the metabolism of probiotics [16]. For example, metabolites derived from *Limosilactobacillus fermentum* have been shown to improve kidney function and histology in chronic kidney disease (CKD) mouse models, along with enhancing antioxidant activity [17].

Natural products contain polyphenols and flavonoids that offer physiological benefits by modulating disease-related pathways [18]. Guava (*Psidium guajava* L.) leaf, used in this study, is known for its antidiabetic [19], antioxidant [20], and anti-inflammatory properties [21]. However, its clinical efficacy is limited by low bioavailability, as only a small portion of the active compounds is absorbed [22]. This requires high intake levels, making it difficult to use effectively.

To address this issue, bioconversion of natural products using probiotics has emerged as a promising approach, enhancing bioavailability of natural products with their metabolites [23]. For example, polyphenols, which are abundant in guava leaf, are often present in glycosidic or esterified forms. These conjugated structures hinder intestinal absorption but simultaneously provide ideal substrates for microbial enzymes such as β-glucosidase and decarboxylase, enabling their transformation into smaller, more bioavailable metabolites. The metabolites may exhibit enhanced bioavailability, improved chemical stability, or even novel bioactivities compared with their parent compounds [24]. Despite increasing interest, research on natural product bioconversion remains limited, particularly in terms of how specific probiotic-natural product combinations influence metabolite generation and efficacy.

Our previous in vitro study showed that GBL exerted significantly stronger antioxidant and antiglycation effects than LF. GBL contains both guava-derived polyphenols (e.g., gallic acid, glucogallin) and bacterial metabolites, offering potential additive and/or synergistic effects [25]. In a T2DM mouse model, GBL improved hepatic energy metabolism and reduced lipid accumulation and inflammation [26]. Based on these findings, both LF and GBL may serve as therapeutic agents for metabolic diseases. However, the effects of GBL on DN remain unexplored.

This study aims to evaluate the potential of GBL to alleviate hyperglycemia-induced renal damage in T2DM mice and elucidate its underlying mechanisms.

## 2. Materials and Methods

### 2.1. Preparation of Bioconverted Guava Leaf Extract

Dried guava leaf (Jeju Island, Korea) was extracted with 50% ethanol and then freeze-dried to yield a powder. For bioconversion, the extract was added to MRS broth at a concentration of 0.5% (*w*/*v*), while MRS broth without extract served as a control. The optical density of *Limosilactobacillus fermentum* KCTC 150720BP was standardized to 1.0 (≈2.02 × 10^9^ CFU/mL) at 600 nm using a spectrophotometer. Bacterial cultures were inoculated into media with or without guava leaf extract, achieving a final inoculum adjusted to 5% (*v*/*v*). After 24 h of incubation, the culture supernatants were collected by centrifugation (11,001× *g*, 10 min, 4 °C). The extracellular metabolites were then isolated from the supernatants and fully dried. The resulting metabolites from media with or without guava leaf extract supplementation were designated GBL and LF, respectively, for subsequent animal studies. Detailed manufacturing methods were described in our previous study [25].

### 2.2. Experimental Design

The induction of T2DM followed the protocols described in earlier research [27]. 4-week-old male C57BL/6J mice (Raon Bio, Yongin-si, Gyeonggi-do, Korea) were housed under controlled conditions (22 ± 1 °C, 50 ± 5% humidity, 12-h light/dark cycle). After a one-week acclimatization period, six mice were assigned to the normal control (NC) group and fed a 10 kcal% fat diet (D12450J; matching sucrose to D12492; Research Diets, New Brunswick, NJ, USA). The remaining mice received a 60 kcal% fat diet (D12492; Research Diets, New Brunswick, NJ, USA) as the diabetic (DM) group. After 4 weeks of diet feeding, DM mice received two intraperitoneal injections of streptozotocin (80 mg/kg BW; Sigma-Aldrich, St. Louis, MO, USA) dissolved in citrate buffer (pH 4.5). NC mice received only citrate buffer. Within 3 weeks following the final injection, mice exhibiting fasting blood glucose (FBG) levels exceeding 300 mg/dL on at least two separate occasions were classified as diabetic.

Following diabetes induction, mice were allocated into 4 groups: (1) NC (*n* = 6), normal control mice; (2) DMC (*n* = 6), diabetic control mice; (3) LF (*n* = 6), diabetic mice supplemented with metabolites of *L. fermentum* (LF) (50 mg/kg BW/day); (4) GBL (*n* = 5), diabetic mice supplemented with metabolites derived from guava leaf extract bioconverted by *L. fermentum* (GBL) (50 mg/kg BW/day). The sample size per group was determined according to standard practice in animal research, assuming a normal distribution [28]. Randomization was used for group assignment, and no statistical differences were observed between groups. LF and GBL were dissolved in distilled water and orally gavaged for 15 weeks. The dose of LF or GBL used in mice was equivalent to a human dose of 4.05 mg/kg [29]. Body weight and food intake were monitored weekly. FBG was measured after 8 h of fasting each week through tail vein sampling using a glucometer (Osang Healthcare, Anyang-si, Gyeonggi-do, Korea). At the end of the study, mice were anesthetized with a ketamine injection. All experimental procedures were conducted under blinded conditions, and no subjects were excluded. Ethical approval was obtained from the Institutional Animal Care and Use Committee of Kyung Hee University [KHSASP-23-153; approved on 15 May 2023], and procedures complied with institutional guidelines.

### 2.3. Hemoglobin A1c Analysis

Hemoglobin A1c (HbA1c) measurement was conducted before sacrifice using the Clover A1c Analyzer (Infopia Co., Ltd., Anyang, Korea) with 4 μL of blood obtained from the tail vein.

### 2.4. Oral Glucose Tolerance Test

The oral glucose tolerance test (OGTT) was conducted following an 8-h fasting period. A 50% glucose solution (2 g/kg BW) was administered through oral gavage. Blood glucose level was determined with a glucometer (Osang Healthcare, Anyang-si, Gyeonggi-do, Korea) at 0, 15, 30, 60, 90, 120, 150, and 180 min. The area under the curve (AUC) for 180 min was calculated based on glucose levels using the trapezoidal method.

### 2.5. Body Composition Analysis

Body composition was analyzed immediately before sacrifice using dual-energy X-ray absorptiometry (InAlyzer, Medikors, Seungnam, Korea). After being anesthetized using ketamine and xylazine injection, each mouse was positioned on the scanning bed with limbs and tail fully extended.

### 2.6. Renal Histological Analysis

Kidney (*n* = 3 per group) was fixed in paraffin and sectioned at a thickness of 4 µm. The sections were stained with hematoxylin and eosin (H&E) to assess renal morphology. The glomerular area and Bowman’s space area were quantified with ImageJ software (ver. 1.54k, NIH, Bethesda, MD, USA). Each value was represented as an average of 20 glomeruli per mouse.

### 2.7. Protein Extraction and Western Blot Analysis

Kidney tissues were homogenized in hypotonic buffer containing protease and phosphatase inhibitors (Thermo Fisher, Waltham, MA, USA). After incubation on ice for 1 h, lysates were centrifuged at 1945× *g* for 10 min at 4 °C. The resulting supernatants were further centrifuged at 9078× *g* for 30 min at 4 °C to obtain the cytosolic fraction. Nuclear pellets were re-suspended in hypertonic buffer, incubated on ice for 1 h, and centrifuged at 9078× *g* for 20 min to yield nuclear extracts [30]. The proteins (*n* = 5~6) were separated by SDS-PAGE gel electrophoresis and subsequently transferred to a PVDF membrane (Millipore, Billerica, MA, USA). The membranes were incubated overnight with the appropriate primary antibodies, followed by exposure to corresponding secondary antibodies. The protein bands were detected using the ECL reagent (Biorad, Hercules, CA, USA), and the band images were captured with a Syngene G box (Syngene, Cambridge, UK) and quantified using GeneTools software (ver. 4.3.16.0, Syngene, Cambridge, UK). All quantifications were normalized to the levels of α-tubulin (cytosol) or Lamin B1 (nucleus) and expressed relative to the NC group.

The following primary antibodies were used: p-AMPK, AMPK, SIRT1, RAGE, Nrf2, catalase, HO-1, MnSOD, NLRP3, ASC, caspase-1, IL-1β, NF-κB, TNF-α, IL-6, MCP-1, iNOS, p53, Bax, Lamin B1 (Cell Signaling Technology, MA, USA, 1:1000); PGC-1α, ICAM-1, Bcl-2, caspase-8, caspase-9, caspase-3 (Santa Cruz Biotechnology, CA, USA, 1:200); α-tubulin (Sigma Aldrich, MO, USA, 1:5000)

### 2.8. Statistical Analysis

Data were expressed as means ± SEM. The statistical significance of differences was determined using one-way ANOVA followed by Duncan’s multiple range test, performed with SPSS software (ver. 23.0, SPSS Inc., Chicago, IL, USA). A value of *p* < 0.05 was considered statistically significant.

## 3. Results

### 3.1. Effects of GBL Supplementation on Body Weight, Body Composition, Kidney Weight, and Food Intake in T2DM Mice

The body weight, body composition, and kidney weight exhibited no significant differences among all groups. However, food intake differed significantly, with the DMC group exhibiting higher consumption than the NC group. In particular, GBL supplementation resulted in a marked reduction in food intake compared to the DMC group (Table 1).

### 3.2. Effects of GBL Supplementation on Glycemic Regulation in T2DM Mice

FBG levels were significantly elevated in all diabetic groups compared to the NC group prior to the administration of any supplements. However, after 10 weeks of supplementation, FBG levels in the LF and GBL groups were significantly reduced compared to those in the DMC group. At the 15th week of supplementation, FBG levels in both treatment groups were statistically comparable to those of the NC group.

Likewise, the HbA1c level of the DM group was higher than that of the NC group. However, after LF and GBL treatments, the elevated HbA1c levels significantly decreased compared to the DMC group (Table 2).

### 3.3. Effects of GBL Supplementation on Glucose Tolerance in T2DM Mice

**Figure 1:**
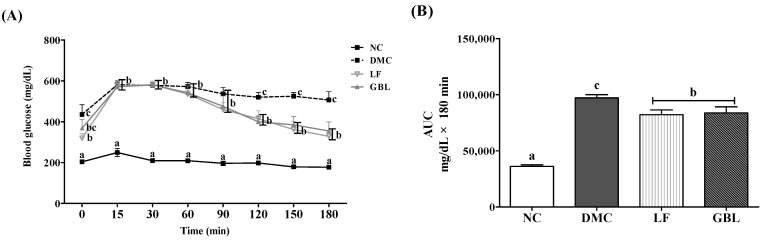
Effects of GBL supplementation on glucose tolerance, (**A**) OGTT and (**B**) OGTT AUC in T2DM mice. Data are expressed as means ± SEM (*n* = 5~6). Means with different letters were significantly different (*p* < 0.05).

A sharp rise in blood glucose levels was observed in the DM group after glucose administration, and a hyperglycemic state persisted throughout the progression of the OGTT. After 120 min, the blood glucose levels of the LF and GBL groups significantly decreased compared to those in the DMC group (Figure 1A).

The AUC of the OGTT was calculated to evaluate the therapeutic efficacy. A significant increase in AUC was noted in the DMC group relative to the NC group, whereas AUC values in the LF and GBL groups were significantly decreased compared with the DMC group (Figure 1B).

### 3.4. Effects of GBL Supplementation on Renal Morphology in T2DM Mice

**Figure 2:**
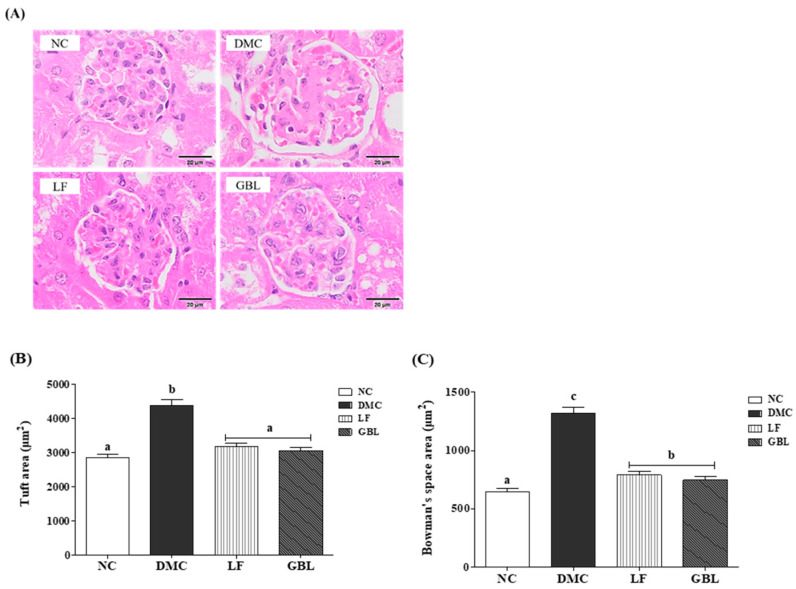
Effects of GBL supplementation on renal morphology, (**A**) H&E staining of renal tissue sections (400× magnification), (**B**) glomerular area, and (**C**) Bowman’s space area in T2DM mice. Data are expressed as means ± SEM (*n* = 3). Means with different letters were significantly different (*p* < 0.05).

Representative renal morphology for each group is shown in Figure 2A. Glomerular surface areas in renal cortical sections were quantified to evaluate the extent of glomerular hypertrophy. A significant enlargement of glomerular structures was observed in the DMC group relative to the NC group, whereas the LF and GBL groups exhibited significantly reduced glomerular hypertrophy (Figure 2B). Bowman’s space was visualized as a narrow, well-defined white line in the NC group. In contrast, it was markedly widened in the DMC group and was reduced in the LF and GBL groups compared with the DMC group (Figure 2C).

### 3.5. Effects of GBL Supplementation on the Renal RAGE in T2DM Mice

**Figure 3:**
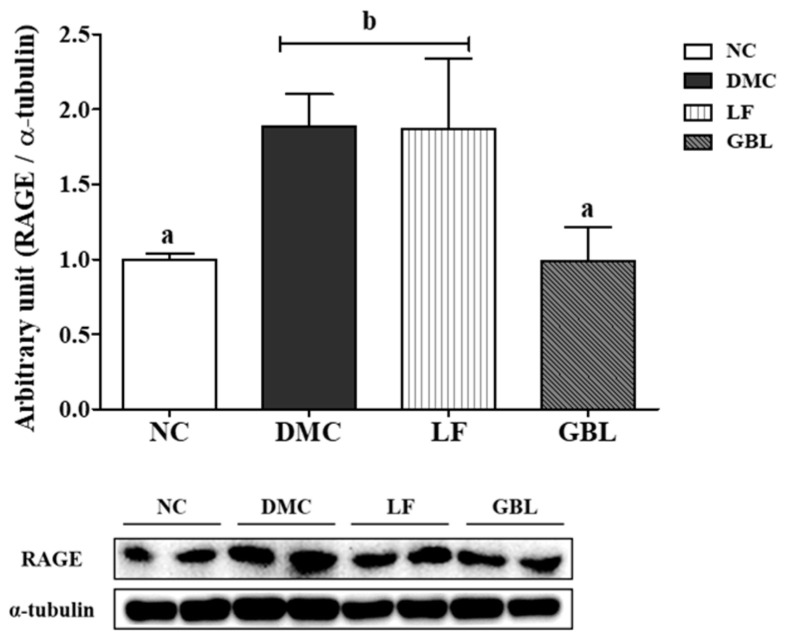
Effects of GBL supplementation on the renal RAGE in T2DM mice. Data are expressed as means ± SEM (*n* = 5~6). Means with different letters were significantly different (*p* < 0.05).

The protein level of the RAGE in the kidney was significantly higher in the DMC group compared to the NC group. The GBL group showed a marked reduction in RAGE expression compared to the DMC group (Figure 3).

### 3.6. Effects of GBL Supplementation on Renal Oxidative Stress in T2DM Mice

**Figure 4:**
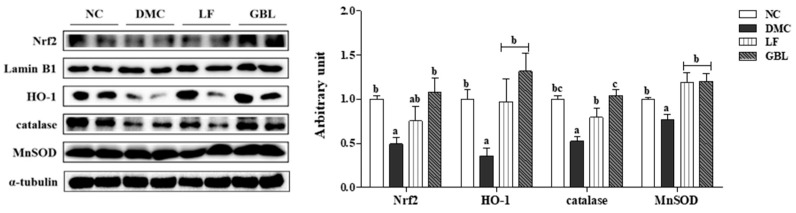
Effects of GBL supplementation on renal antioxidant defense system markers (Nrf2, HO-1, catalase, and MnSOD) in T2DM mice. Data are expressed as means ± SEM (*n* = 5~6). Means with different letters were significantly different (*p* < 0.05).

The protein level of Nrf2 was significantly reduced in the DMC group compared to the NC group. However, the protein level of Nrf2 in the GBL group was significantly elevated relative to the DMC group. Similarly, the protein levels of HO-1, catalase, and MnSOD, which are downstream antioxidant enzymes of Nrf2, were reduced in the DMC group compared to the NC group, but were increased in both the LF and GBL groups relative to the DMC group (Figure 4).

### 3.7. Effects of GBL Supplementation on Renal Inflammation in T2DM Mice

**Figure 5:**
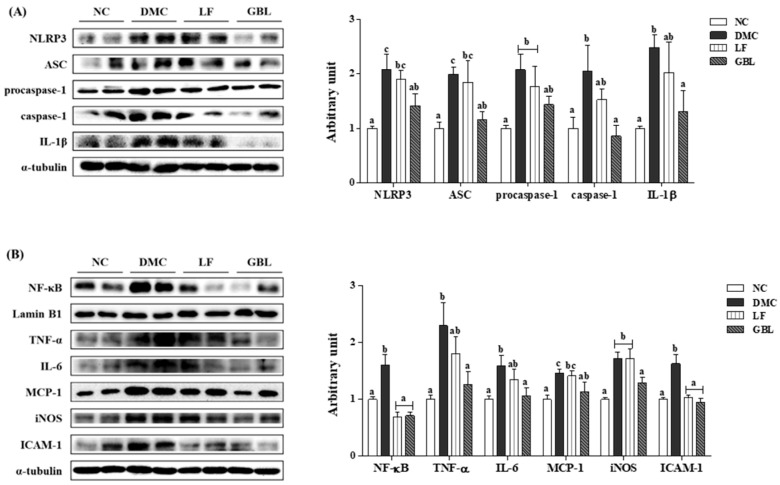
Effects of GBL supplementation on renal inflammation-related markers, (**A**) NLRP3 inflammasome (NLRP3, ASC, procaspase-1, caspase-1, and IL-1β) and (**B**) markers of pro-inflammatory response (NF-κB, TNF-α, IL-6, MCP-1, iNOS, and ICAM-1) in T2DM mice. Data are expressed as means ± SEM (*n* = 5~6). Means with different letters were significantly different (*p* < 0.05).

The protein levels of NLRP3 inflammasome components, as well as downstream effectors, were significantly increased in the DMC group compared to those of the NC group. In contrast, the protein levels of NLRP3, ASC, caspase-1, and IL-1 were significantly decreased in the GBL group relative to the DMC group (Figure 5A).

The protein level of NF-κB was markedly elevated in the DMC group compared to the NC group, but was significantly reduced in both the LF and GBL groups relative to the DMC group. Consistent with the pattern of NF-κB expression, the protein level of ICAM-1 was elevated in the DMC group compared to the NC group and was significantly decreased in the LF and GBL groups relative to the DMC group. Notably, the elevated protein levels of TNF-α, IL-6, MCP-1, and iNOS in the DMC group were attenuated only in the GBL group (Figure 5B).

### 3.8. Effects of GBL Supplementation on Renal Apoptosis in T2DM Mice

**Figure 6:**
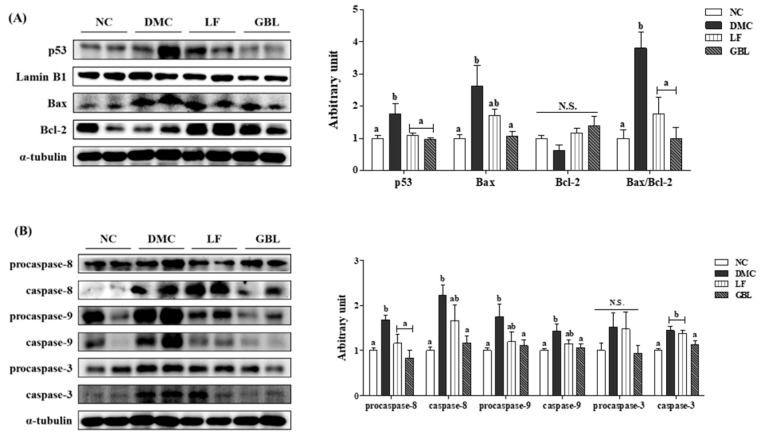
Effects of GBL supplementation on renal apoptosis-related markers, (**A**) p53, Bax, Bcl-2, (**B**) procaspase-8, caspase-8, procaspase-9, caspase-9, procaspase-3, and caspase-3 in T2DM mice. Data are expressed as means ± SEM (*n* = 5~6). Means with different letters were significantly different (*p* < 0.05). N.S., not significant.

The protein level of p53, a transcription factor involved in apoptosis, was significantly elevated in the DMC group compared to the NC group. However, supplementation of LF and GBL markedly decreased p53 expression, bringing it closer to the level observed in the NC group. Similarly, the protein level of Bax was significantly increased in the DMC group, whereas GBL supplementation significantly decreased Bax expression relative to the DMC group. The expression of Bcl-2 showed no significant differences among the experimental groups. However, the Bax/Bcl-2 ratio, a key indicator of pro-apoptotic activity, was significantly lower in the LF and GBL groups compared to the DMC group (Figure 6A).

Additionally, the protein levels of procaspase-8, caspase-8, procaspase-9, caspase-9, and caspase-3 were significantly upregulated in the DMC group compared to the NC group, indicating enhanced apoptotic signaling. Treatment with GBL significantly reduced these markers. Notably, the elevated procaspase-8 levels in the DMC group were effectively diminished in the LF and GBL groups. In contrast, procaspase-3 levels remained consistent across all experimental groups, showing no significant changes (Figure 6B).

### 3.9. Effects of GBL Supplementation on Renal Energy Metabolism in T2DM Mice

**Figure 7:**
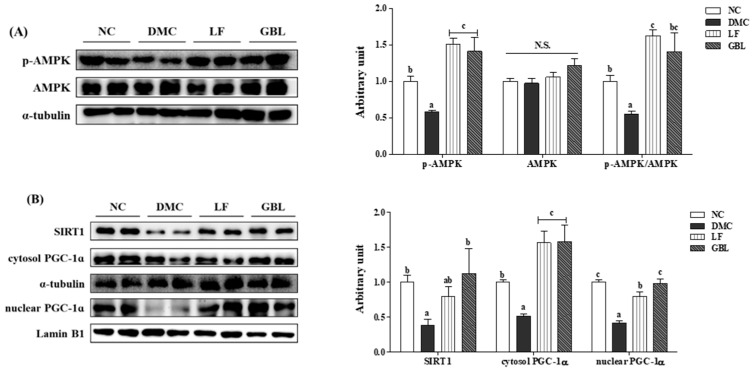
Effects of GBL supplementation on renal energy metabolism-related markers, (**A**) phosphorylation of AMPK (p-AMPK and AMPK) and (**B**) SIRT1 and PGC1-α in T2DM mice. Data are expressed as means ± SEM (*n* = 5~6). Means with different letters were significantly different (*p* < 0.05). N.S., not significant.

The protein level of AMPK was not significantly different among the experimental groups. The protein level of p-AMPK was significantly lower in the DMC group relative to the NC group, but it was significantly elevated in the LF and GBL groups compared to the DMC group. Consequently, the p-AMPK/AMPK ratio in the LF and GBL groups was significantly elevated relative to the DMC group (Figure 7A).

Furthermore, the protein level of SIRT1 was significantly reduced in the DMC group compared to the NC group, but it was elevated in the GBL group compared to the DMC group. Consistent with these results, the protein level of PGC-1α significantly declined in the DMC group compared to the NC group, regardless of whether it was located in the nucleus or cytosol, while it was markedly increased in the LF and GBL groups compared to the DMC group (Figure 7B).

## 4. Discussion

In this animal study, GBL demonstrated superior efficacy in alleviating hyperglycemia-induced DN in diabetic mice. It markedly improved hyperglycemia, glucose tolerance, and morphological alterations while activating the AMPK/SIRT1/PGC-1α pathway and enhancing antioxidant activity. Notably, the regulatory effect of GBL on NLRP3 inflammasome and NF-κB-driven inflammation, as well as apoptosis, highlights the therapeutic potential of GBL as a multi-mechanistic intervention against DN. These results provide mechanistic insights into the renoprotective actions of bioconverted guava leaf extract, serving as a basis for further therapeutic exploration.

Chronic hyperglycemia is a hallmark of diabetes and a major contributor to glucotoxicity, which induces oxidative stress and inflammation in multiple organs, including the kidney [31]. Both LF and GBL supplementation effectively lowered FBG and improved glucose tolerance, with GBL demonstrating a greater reduction in HbA1c, suggesting superior control of long-term glycemic status. Previous studies have shown that metabolites of *L. fermentum* exert antidiabetic effects in diabetic models. Among them, branched-chain hydroxy acids, such as 2-hydroxyisocaproic acid and 2-hydroxyisovaleric acid, have been reported to enhance glycemic control by improving glucose uptake in muscle cells and suppressing hepatic gluconeogenesis [25,32]. Similarly, postbiotics derived from *L. plantarum* LRCC5314 improved FBG, glucose tolerance, and insulin sensitivity in T2DM mice, supporting the role of probiotic-derived metabolites in modulating glucose metabolism [33]. Taken together, these findings suggest that bacterial metabolites from *L. fermentum* contribute to glycemic improvement in T2DM. Moreover, the overall efficacy of *L. fermentum*-derived metabolites appears to be enhanced when combined with guava leaf via bioconversion, as evidenced by the superior metabolic outcomes observed with GBL supplementation.

Although hyperglycemia contributes to both structural and functional deterioration of the kidney in T2DM, no significant differences in renal function markers were found in this study (data not presented). This may reflect the early stage of DN, where compensatory glomerular hyperfiltration often masks functional decline [34]. Indeed, structural abnormalities such as glomerular hypertrophy and Bowman’s space expansion are known to precede measurable impairments in glomerular filtration [35,36]. Despite the absence of functional improvement, LF and GBL supplementation significantly ameliorated renal morphological alterations, suggesting protective effects on glomerular architecture under diabetic conditions. Similar findings have been reported for *L. fermentum*-derived postbiotics in a cisplatin-induced CKD model [17]. The observed structural improvements suggest that GBL, like LF, may help delay the onset of renal functional decline by preserving glomerular morphology. This effect might be mediated by upstream modulation of diabetes-related pathological pathways, including oxidative stress, inflammation, apoptosis, and energy metabolism.

With respect to the energy metabolism, mitochondrial dysfunction and impaired energy homeostasis are central contributors to DN, leading to oxidative stress, inflammation, and apoptosis [37]. The AMPK/SIRT1/PGC-1α axis plays a critical role in maintaining mitochondrial integrity and energy balance, but is typically downregulated under diabetic conditions [38]. Prior investigations reported that probiotic strains such as *L. plantarum* or its heat-killed derivatives improved metabolic dysfunction in rodent models by activating AMPK and modulating the SIRT1/PGC-1α pathway [39]. In the present study, both LF and GBL increased renal PGC-1α expression and the p-AMPK/AMPK ratio in T2DM mice, while only GBL significantly upregulated renal SIRT1 expression. Consistent with our previous hepatic findings, GBL more effectively restored the renal AMPK/SIRT1/PGC-1α axis compared to LF in T2DM mice. This enhanced activation may underlie the broader metabolic benefits of GBL across multiple organs affected by T2DM and contribute to its renoprotective effects under hyperglycemic conditions.

Chronic hyperglycemia activates the AGE–RAGE axis, promoting oxidative stress and inflammation at the molecular level [7]. This study showed that GBL supplementation significantly suppressed renal RAGE expression, whereas LF had no such effect, suggesting that GBL more effectively inhibits AGE–RAGE signaling under hyperglycemic conditions. Suppression of RAGE activation was accompanied by restoration of the oxidative stress counteracting pathway [8]. Notably, only GBL restored renal Nrf2 expression, a master regulator of cellular antioxidant responses. In line with this, both LF and GBL upregulated Nrf2-dependent antioxidant enzymes, including HO-1, catalase, and MnSOD, with GBL exhibiting a more substantial effect. The enhanced antioxidant effects of GBL may be attributed to guava leaf-derived polyphenols in GBL. Gallic acid and glucogallin have been shown to downregulate RAGE and attenuate AGE-induced oxidative damage in models of cardiac remodeling [40]. In addition, *L. fermentum*-derived postbiotics have demonstrated antioxidant effects in CKD models, including reductions in ROS and lipid peroxidation [17]. Thus, these findings suggest that GBL alleviates oxidative stress more effectively than LF by modulating both AGE–RAGE and Nrf2-dependent pathways.

Inflammatory responses and immune modulation in DN are driven, in part, by oxidative stress–mediated activation of redox-sensitive pathways, such as NF-κB and the NLRP3 inflammasome [41]. In the present study, GBL treatment effectively suppressed the expression of renal NLRP3, ASC, and caspase-1, leading to a reduction in IL-1β production in T2DM mice. Renal NF-κB expression and pro-inflammatory cytokine and chemokine levels were also notably suppressed in the GBL group, reflecting its pronounced capacity to counteract hyperglycemia-induced renal inflammation. The superior anti-inflammatory activity of GBL may be attributed to its distinct metabolite profile, including guava leaf–derived flavonoids and microbe-derived compounds. Bioactive flavonoids, such as catechin and quercetin, exert anti-inflammatory effects in part by modulating immune responses [42,43]. The combined actions of metabolites may exert additive and/or synergistic effects, further strengthening the anti-inflammatory efficacy of GBL. Collectively, the dual targeting of NF-κB and NLRP3 inflammasome signaling by GBL highlights its mechanistic basis for renoprotection in diabetic conditions and supports its potential as a therapeutic strategy for DN.

Apoptotic cell death plays a pivotal role in the progression of DN, particularly through the loss of podocytes and tubular epithelial cells [44]. In this study, GBL supplementation markedly attenuated renal apoptosis by downregulating p53 expression and restoring the Bax/Bcl-2 ratio toward anti-apoptotic signaling. In parallel, SIRT1 levels were upregulated in the GBL group, suggesting its involvement in suppressing p53 activity and promoting cell survival [45]. Furthermore, only GBL effectively inhibited the activation of caspase-8, -9, and -3, indicating suppression of the intrinsic and extrinsic apoptosis cascades [46]. The superior anti-apoptotic efficacy of GBL may be attributed to metabolites, such as asiatic acid, a terpenoid generated through the bioconversion of guava leaf extract, which is absent in LF. A previous study reported that asiatic acid protects against cisplatin-induced acute kidney injury by inhibiting inflammation and modulating apoptotic regulators [47]. These findings suggest that the bioconversion of guava leaf by *L. fermentum* enhances the bioactivity of key metabolites, enabling GBL to target multiple apoptotic mechanisms and better preserve renal structure and function under hyperglycemic conditions.

Taken together, GBL demonstrated comparable benefits to LF in improving glucose metabolism and reducing oxidative stress, but exhibited superior efficacy in suppressing inflammation and apoptosis in DN. The enhanced renoprotective properties of GBL are likely attributable to the unique metabolite composition generated through microbial bioconversion of guava leaf. The results highlight the therapeutic effects of GBL as a targeted strategy for protecting against hyperglycemia-induced renal damage in T2DM. This study was conducted exclusively in male mice to minimize variability related to hormonal fluctuations. Therefore, future research should include both male and female subjects to comprehensively evaluate sex-specific responses. Moreover, long-term studies are necessary to examine the durability of GBL’s protective effects and to further elucidate the underlying molecular mechanisms. Although these preclinical findings are promising, successful clinical translation of GBL will require additional investigations focusing on dose optimization, long-term safety, and differential effects across stages of disease progression.

## 5. Conclusions

The current study demonstrated that GBL effectively alleviates hyperglycemia, glucose tolerance, and abnormal morphological changes in DN. Furthermore, GBL exhibited beneficial effects on the AMPK/SIRT1/PGC-1α-mediated energy metabolism and improved oxidative stress signaling in DN. In particular, GBL is more effective than LF in attenuating renal damage by improving inflammation and apoptosis via NLRP3 inflammasome and NF-κB activation. Conclusively, given the additive and/or synergistic effects of bioconversion with guava leaf, GBL might be a promising nutraceutical in mitigating hyperglycemia-induced renal inflammation and apoptosis in diabetic mice.

## Figures and Tables

**Table 1:** Effects of GBL supplementation on body weight, body composition, kidney weight, and food intake in T2DM mice.

	NC (*n* = 6)	DMC (*n* = 6)	LF (*n* = 6)	GBL (*n* = 5)
Body weight (g)	33.17 ± 1.29	36.56 ± 1.78	33.94 ± 1.21	34.50 ± 2.22
Body composition (%)				
fat mass	24.93 ± 1.25	31.45 ± 2.45	25.97 ± 1.32	29.03 ± 3.13
lean mass	72.39 ± 1.23	66.17 ± 2.40	71.60 ± 1.32	68.59 ± 3.04
Kidney weight (% BW)	0.501 ± 0.011	1.499 ± 0.019	0.532 ± 0.012	0.502 ± 0.030
Food intake (kcal/day)	12.65 ± 0.25 ^a^	14.24 ± 0.31 ^b^	14.84 ± 0.52 ^b^	13.12 ± 0.09 ^a^

Data are expressed as means ± SEM (*n* = 5~6). Means with different letters were significantly different (*p* < 0.05).

**Table 2:** Effects of GBL supplementation on glycemic regulation in T2DM mice.

	NC (*n* = 6)	DMC (*n* = 6)	LF (*n* = 6)	GBL (*n* = 5)
FBG (mg/dL)				
Week 0	173.50 ± 9.25 ^a^	484.00 ± 10.97 ^b^	437.17 ± 25.53 ^b^	443.40 ± 37.30 ^b^
Week 5	168.33 ± 10.47 ^a^	382.17 ± 23.87 ^c^	279.83 ± 26.53 ^b^	330.20 ± 30.94 ^bc^
Week 10	194.17 ± 7.73 ^a^	450.83 ± 55.72 ^c^	306.50 ± 37.97 ^b^	317.80 ± 28.17 ^b^
Week 15	188.50 ± 12.74 ^a^	433.33 ± 50.28 ^b^	281.33 ± 31.08 ^a^	248.80 ± 14.81 ^a^
HbA1c (%)	4.08 ± 0.08 ^a^	7.35 ± 0.30 ^d^	5.68 ± 0.14 ^c^	4.82 ± 0.19 ^b^

Data are expressed as means ± SEM (*n* = 5~6). Means with different letters were significantly different (*p* < 0.05).

## Data Availability

The data presented in this study are available on request from the corresponding author. The data are not publicly available due to privacy. The studies do not involve humans.
